# Opening Up of Editorial Activities at Chemistry Journals. What Does Editorship Mean and What Does It Involve?

**DOI:** 10.3389/frma.2022.747846

**Published:** 2022-03-24

**Authors:** Marianne Noel

**Affiliations:** LISIS, CNRS, INRAE, Université Gustave Eiffel, UMR9003 Laboratoire Interdisciplinaire Sciences, Innovations, Sociétés (LISIS), Marne la Vallee, France

**Keywords:** scientific publishing, editorship, American Chemical Society, Nature Research, chemistry journals

## Abstract

The article unpacks the publishing practices and focuses on the curating work carried out by the editors of chemistry journals. Based on a qualitative analysis of multiple sources in two publishing houses (the American Chemical Society, ACS and Nature Research), it first shows that the role of editor-in-chief covers a wide range of realities and is far from being limited to that of a gatekeeper (the most common metaphor in the literature). In journals that are part of the Nature Research portfolio, in-house editors, who are no longer active scientists, work full time for the journals. The article describes the professional trajectories and skills required to join the publishing house. Interviews highlight collective identity-based actions, attention to the growth and the flow of manuscripts, but also specific epistemic properties of outputs in chemistry. Besides tasks that editors outline “as really the same as they were 100 years ago,” as they spend most of their time handling manuscripts and providing quality assurance, they also travel to conferences to support journals and encourage submissions, visit labs where researchers pitch their work or ask questions about journals, and “educate the actors themselves” about new fields. In both cases studied, the publishing houses partner with institutions to offer events (ACS on Campus programme, *Nature* masterclass) that a university or department can freely host or buy, where editors organize workshops on all aspects of manuscript preparation. Second, publishing houses, whether non-for-profit or commercial, have embraced a catalog logic, where the journals are not necessarily in competition and have an assumed place and hierarchy. At Nature Research, editors-in-chief head business units inscribed in the company's organization. Despite standardized processes imposed by the procedural chain, there is still room to maneuver in these relatively autonomous structures that are ultimately evaluated on their results (the annual production of a certain number of high-quality papers). On the other hand, ACS is seen as a vessel whose course cannot easily be deviated. The conclusion calls for extending this type of investigation to other contexts or types of journals.

## Background and Context

### The Gatekeeping Function of Editors

One of the most common narratives in the scientific literature, whether in information science, sociology, or management, positions the scholarly editor as a gatekeeper. This metaphor, which refers broadly to the process of controlling information as it moves through a gate or filter, has influenced the perception of the potential role of editors, both in journalism and in scientific publishing. In her critical review, Barzilai-Nahon (2008) defines editorial gatekeeping as a critical information role, seen as a way of “orchestrating influence.” Rather than acknowledging the various ways in which the editors' activities are performed, the use of this metaphor suggests that editors simply maintain academic boundaries and power structures, thereby exercising considerable control over scientific discourse. But qualitative studies of editorial gatekeepers are scarce, despite the central role played by these actors.

Many scientometric studies have described the scholarly communication landscape from the perspective of editorial gatekeepers. As Cabanac (2012) has pointed out, Crane (1967) was an early adopter of the term gatekeeper. Inspired by the term coined by political scientist Alfred De Grazia, she empirically confirmed the possibility of an “establishment” in scientific disciplines. Both De Grazia and Crane referred to editors of journals as “the” gatekeepers of science. Many studies of the structure of various scientific disciplines ensued, consisting in analyzing the editorial boards of their most influential journals. Chemistry journals were among the first scrutinized, in the early 1980s (Braun and Bujdosó, 1983; Braun and Dióspatonyi, 2005). With the development of network analysis instruments and of the field of scientometrics itself, studies using quantitative techniques proliferated. They examined the relationship between the scientific achievement of editorial board members and the prestige of journals with which they are associated. Most of these studies are based on the assumption that all of the agents and actions described above can be viewed as interdependent rather than as autonomous units; the actions can then be considered as relational ties (linkages) between agents. Baccini and Barabesi (2010) documented the “interlocking editorship” phenomenon, which refers to a gatekeeper sitting on several editorial boards. Exploring the networks of editors, authors and co-citations in three fields (statistics, economics, and information and library sciences), they then suggested that intellectual proximity is also proximity among authors and among editors of the journals (Baccini et al., 2020). They also affirm that the map of editorial power, the map of intellectual proximity, and the map of author communities tell similar stories. Several examples of nepotism, either relatively older (El Naschi) or recent (Raoult), show that the takeover of a journal by an individual can facilitate the publications of a group of researchers (Gingras and Khelfaoui, 2021). In the wake of the open science movement, a new line of research entitled “editormetric research” has emerged (Mendonça et al., 2018), with new initiatives such as The Open Editors project (Pacher et al., 2021) that collects data about academic journal editors on a large scale and structures them into a single dataset, starting from the fact that much of the current editorial data remain unstructured and have to be collected manually.

The question underlying this article is: what is lost when editorship is reduced to gatekeeping? This metaphor creates distance between editors and academics. Positioning editors as a group notably distinct from colleagues or friends in academia, it categorizes them as administrators allowing for privileged access to publishing platforms, thus maintaining academic power structures.

Although academics are quite talkative about their editorial work (Stang, 2003; Zedeck, 2008; Wise, 2018; Chibnik, 2020), there is little empirical work on editorship (Glonti et al., 2019). Even though a growing number of studies on peer reviewing focus on how publication decisions are taken (Fyfe et al., 2020; Kaltenbrunner et al., 2021), very few studies have examined the conditions of the editorial production of journals, and even less so of articles. A noteworthy exception is the ethnographic research carried out by Serge PJM Horbach at the editorial office of two large academic publishers (Horbach, 2020; Horbach and Halffman, 2020). Based on 41 interviews or individual meetings and 10 group meetings, the structure and role of the editorial process at big publishers are described in great detail. The authors highlight a very layered and hierarchical publication process that organizes the editorial process in a long procedural chain, with highly specialized division of labor.

Last but not least, the contribution of historians of periodicals on editorship is absolutely essential. First, they argue that journals have never been a passive vessel, and that instead, they have been a site where the rules of science themselves were debated and developed (Baldwin, 2015; Fyfe et al., 2017; Csiszar, 2020; Tesnière, 2021). They strongly argue that a better understanding of the journal's past is crucial to imagining future forms of knowledge expression and organization. Interestingly, the digitization of past issues of academic journals has focused exclusively on content, neglecting the front and back pages and thereby erasing the whole editorial team from the digital records of the past (Vermeir, 2020). Issued in 2020, the special issue in *Centaurus* (see the introduction by Fyfe and Gielas, 2020) explored the rich variety of editorial processes and strategies used in different places, contexts, and times (1750–1950). Placing this research in the lineage of historical works allows us to put the importance of current changes into perspective, notably because the proposed study focuses on two publishers who have a long trajectory in scientific publishing.

### A Focus on Chemistry

Although there are historical monographs or articles on journals such as *Nature* (Baldwin, 2015), *Astronomy & Physics* (Pottasch, 2011), *Chemistry: a European Journal* (Noel, 2019) and so on, few qualitative works have recently taken a discipline as the analytical operator. The reasons are diverse: disagreements around the epistemologies contained in the disciplinary or inter(-anti-) disciplinary perspectives induced a pendulum movement in STS studies. Yet disciplines constitute the educational units of organization in universities. They are the practitioners' stable reference framework, providing the intellectual and material resources, language, and questions (Marcovich and Shinn, 2014: 172).

As they are called today, the chemical sciences are broad and include a wide range of research topics, from the basic sciences to highly applied research domains. They are widely represented in American universities (there are nearly 700 chemistry departments, of which more than 200 offer a PhD training programme) and elsewhere, and account for just over 4% of federal R&D spending in the United States. In the elite universities where I carried out my research (in France, Switzerland and the USA), the chemistry workforce accounts for about 10% of the total faculty. Journal articles are, with patents, the main type of publication in chemistry. These journals are purchased by university libraries and public and private R&D centers. There are therefore several thousand credit-worthy buyers—an important market for these publications.

Chemistry is a good example of how modern research is organized in an impressive array of sub-disciplines and hybrid-discipline formations (Meinel in Reinhardt, 2001: IX). The epistemic profile of the discipline is shaped by the various “fields” where chemists are working. Chemistry is organized into a large and loose rhizome network (Bensaude-Vincent, 2018) that has given rise to a wide editorial panorama.

As Cronin et al. (2004) have argued, in chemistry the dominant model of scientific knowledge production is industrial rather than artisanal in character. Using articles published, Rosenbloom et al. (2015) documented a rapid acceleration in the rate at which chemical knowledge was produced in the late 1990s and early 2000s relative to the financial and human resources devote to its production. Today, the *Journal of the American Chemical Society* (*JACS*), of which it is the “flagship” journal, is a periodical that produces more than 19,000 articles a year. In 2021, the editorial team of *Chemistry of Materials* announced that it has succeeded to manage up to 20,000 articles over their entire time of service (Toro and Skrabalak, 2021).

Not only authors, readers and editors, but also librarians navigate in this crowded and stratified landscape. From a Web of Science query, Larivière et al. (2015) estimate that, in 2015, 70% of the articles produced in chemistry were available through ACS journals. The figure may seem high but it gives an idea of ACS hegemony in the chemical publishing sector. However a recent study conducted from the analysis of a corpus extracted from Ulrich's commercial database shows that nearly 2,300 chemistry journals have been active since 2001 (Noel and Bordignon, 2021).

From a historical perspective, chemistry has built its system of publications on professional norms, standards and conventions and that relies—especially in the United States—on the development of a learned society (the American Chemical Society, ACS), which is not a commercial corporation. ACS journals are embedded within communities. Unlike the (British) Royal Society (Fyfe, 2020), the ACS took the turn of internationalization without too much hindrance in the 1950–60s, notably because special attention was paid to its international authors (Noel, 2020).

In the past decades, digitization has been attended by commercialization, with an increasingly concentrated journal market led by a handful of large, for-profit publishing houses (Strasser and Edwards, 2016; Fyfe et al., 2017). But chemistry has remained attached to journals governed by its learned societies, be it the ACS (its portfolio consists of around 50 journals) or the British Royal Society of Chemistry (around 100). Other publishers covering chemistry include Wiley, whose chemistry titles are co-owned by a collection of European societies (EuChemS), Elsevier, and so on. Nature Research is a relative newcomer to the field. This makes chemistry an interesting case to study.

## Analytical Framework

What are the contemporary conceptions of chemistry editing? The dominant position of the “elite” journals is regularly questioned in the field of STM publishing. In the salvo of criticism against the publication system that accompanied the development of open access, renowned scientists, often through the press or opinion letters, have attacked Nature Research journals on the pretext that they publish only the most “flashy” research. In a letter to The Guardian (Schekman, 2013), Randy Schekman, Nobel Prize-winning biologist, said his lab would no longer send papers to the top-tier journals, *Nature, Cell*, and *Science*. Schekman is the editor of *eLife*, an online journal set up by the Wellcome Trust, which is a competitor to the other three. He claimed that pressure to publish in “luxury” journals encouraged researchers to pursue trendy fields of science instead of doing more important work. The problem is exacerbated, he added, by editors who are not active scientists but professionals who favor research subjects that are likely to make a splash, and “accept papers that will make waves because they explore sexy subjects or make challenging claims.”

Khelfaoui and Gingras (2020, 2021) developed the idea that academic publishers that engage in scholarly journal branding, whether in the form of product line or brand extensions, contribute to the transformation of the scientific “community” into a scientific market. Based on a review of the strategies available on the web, they propose a detailed analysis of the “pioneering and thus paradigmatic case of *Nature”* which they extend to other publishers (whether commercial such as Frontiers Media, not-for-profit such as PLOS, or run by not-for-profit scholarly societies such as the American Association for the Advancement of Science, AAAS or the ACS). Considering that this branding strategy extended to all publishers has been pursued particularly aggressively over the past 5 years, they argue that “the dynamic of the competition in the scientific field is now responding more to the logic of market than to that of a community.” For these authors, the publishing field is a field of forces that publishers try to turn to their advantage to derive the monetary or symbolic benefits associated with it. They use Bourdieu's model of capital conversion, claiming that publishers turn a specific form of capital, namely the symbolic capital of their high-profile publications, into economic capital. Khelfaoui and Gingras argue that the newly created journals benefit from the name recognition and reputation of the originals after which they are named. Plus, through a manuscript routing mechanism, the publishers redirect papers refused by the prestigious journals to the less prestigious ones of the brand (the so-called “derivative journals”) or to one of the lower-impact journals on their list, which may require an article processing charge for publication (a model where the authors/institutions pay fees to have the electronic versions of their articles in open access).. Bourdieu's relational sociology heavily emphasizes the domination and power relations, notably symbolic.

In the same vein, many arguments in the literature underline a convergence across countries, disciplines, institutions, and so on: globalization as the top-down diffusion of global templates, the rise of new modes of measuring and evaluating academic work, etc. But they neglect the ways in which academic actors themselves are involved in publishing situations. Moreover, equating a firm and a learned society (that Khelfaoui and Gingras labeled a “private non-profit organization”) overlooks the fact that scholarly societies are an integral part of academic research and teaching institutions (Paradeise and Thoenig, 2013). While the ACS is the owner of a heritage/patrimony built up collectively over a long period of time (the Treasurer & CFO Division oversees the society's annual operating budget of $550 million (2017) and the management of $1.7 billion of investments), it is not required to provide a return on investment to pension funds and venture capital firms who now run most of the large private publishing houses.

As mentioned earlier, academic work that specifically takes chemistry journals as its research object is rare. Interestingly, Volkmann et al. (2014) have developed the idea of a chemistry journals market operating as a “strategic action field,” a social space populated both by “incumbents” (which clearly dominate the field) and “challengers” (Fligstein and McAdam, 2011). In order to contrast the two worlds of academic publishing in chemistry and German sociology, they conducted qualitative interviews with editors and publishers of five chemistry publishing houses and took a “very-large worldwide operating publisher with regard to chemistry publications,” which is a business firm, as in-depth case study. The analytical framework they heuristically proposed allows us to study a situation where windows of opportunities have opened up, leading to the entry of new players into chemistry publishing over the past decades. These challengers (i.e., chemistry journals at Nature Research) are the first target group of my study. Action fields are bounded, and the actors are strongly interdependent and share a common understanding of the stakes and rules of the field. In this framework, the skilled social actors are at the heart of the emergence of new fields (Fligstein, 2001). The early work of Bergmann (1965), complemented by that of Ottolini (2020), highlights the decisive role of the skills developed by actors and administrations in institutional changes in academia.

By borrowing from STS, from organization studies and the existing literature on the process of economization, I propose to complete this approach by describing the market of chemistry journals from an analytical framework which is not that of the meeting of a supply and a demand. As emphasized by Musselin (2018: 658) in her study of new forms of competition in higher education, the term “market” should only be applied to situations where competition and exchange are simultaneously present. Markets are here understood as devices for qualifying goods and calculating their value (Callon and Muniesa, 2005). The actor-network theory conceives of collectives in a manner quite different from a Bourdieusian approach, by populating them with humans and non-humans and, above all, by emphasizing the collective in the process of being made, on associations and not on an already existing “social.” Michel Callon has conceptualized economic actors as constituted of socio-technical agencements: collectives of human beings, technical devices, algorithms, and so on (Hardie and MacKenzie, 2007). For Callon, a focus on market agencements places innovation as the driving force of market evolution, instead of just price adjustments (Callon, 2017).

In this work, the journal is both a set of copies and a “collection” of articles, a material object (with a required number of pages or words, a format to be complied with, a publication date to respect etc.) and a social organization that creates that object. Like other cultural goods or professional services, the academic journal is part of an economy of singularities (Karpik, 2007). Singularities are multidimensional and indivisible goods and services, characterized by their symbolic value and uncertainty as to their quality. Offering a narrative that starts from editors' testimonies in a relational perspective, I take the chemistry journals and their registration in the commercial space as the main focus of study and critically examine the modalities of market coordination among actors.

The remainder of this article is structured as follows: in the section “Editors' key tasks, respective role and relationships with other,” I first present the professional trajectories and skills of editors-in-chief required to join the publishing houses. I then look at the modalities of work and organization of the editorial process in both working environments. The next section “Hierarchies and dependencies within publishing houses” illustrate that editors are aware of hierarchies and dependencies within the respective publishing houses, carry out their activities under production and deadlines constraints and have to navigate between different types of request that come from authors or other publishers' organizational units (whether be journals, regionally oriented magazines such as *Nature Asia*, database of author affiliation information such as *Nature Index*, etc.) and so on. I also introduce the recent debates on the future of chemistry journals: emerging of the figure of curator (in the primary sense derived from the Latin cura, meaning “to take care”), attention paid to the inclusion of young researchers in decision-making bodies, distancing of metrics and indicators. In the discussion part, I broaden the scope of the previous results by looking at the general mechanisms at play between chemistry journals that operate within disciplinary-oriented organizational and identity logics.

## Approach, Methods, and Structure of the Article

### Approach Chosen

One of the aims of this article is to explore the wide range of realities covered by the functions of editors-in-chief of chemistry journals. The term “editorship” as conceived by Shattock (1983) encompasses the activities of defining the goals and scope of a journal and ensuring that new issues appear regularly, but it also refers to a role that is meaningful in the scientific community and, more broadly, in the academic world. As Fyfe and Gielas have pointed out (2020), editorship has received rather less scholarly attention to date than authorship or peer review. My stance in this article is both to properly complicate and clarify this important position in the scholarly publishing. Moreover, the variety of the titles encountered in the fieldwork or in the job offers (editor-in-chief, chief editor, in-house editor, junior editor, senior editor, associate editor, copy editor, etc.) argues for investigation of the tasks carried out by editors in concrete terms.

Using a sociological approach (i.e., the sociology of professions and occupational groups understood in the sense of Demazière and Gadéa (2009) as an evolving, unstable and open process) favoring a relational perspective (a chemistry journals market described as a market agencement), I examine what editorship means in chemistry journals and what it involves, considering the importance of the management of the complex mechanisms that lie behind the editorial process.

Who are the editors-in-chief, what are their career paths? How do they build their skills, their expertise and know-how and their professional ethics? Are their skills adapted to these missions, and do they need to evolve? How and by whom are they evaluated?

Under what conditions do they carry out their missions within organizations on a daily basis and in concrete terms? To what extent are they likely to influence and determine the way in which the economy of scientific publication is structured?

Based on an exploration of editors-in-chief practices, this empirical study is also a means of testing whether the categorization “for profit”/“not-for-profit” is operational when moving from one publishing house to another. By contrasting two publishers, I look for similarities rather than broad explanatory categories.

### Presentation of the Two Publishing Houses

Roughly speaking, one of the key features that distinguish the two environments is that the Nature Research journals and their associated “specialty” titles have full-time professional editors^1^ in their offices (for the London office, several floors of open space) working on “their” journals. By contrast, the vast majority of ACS journals have academic editors who work off-site at a university or research institution, and work on the journal in their spare time. I have often come across this distinction (academic or professional) in the representations that academics have of editorship.

Nature Research is a division of the international scientific publishing company Springer Nature that publishes academic journals, magazines, online databases, and services in science and medicine (including Nature Masterclasses and Naturejobs) (Inchcoombe, 2016).

**Figure 1:**
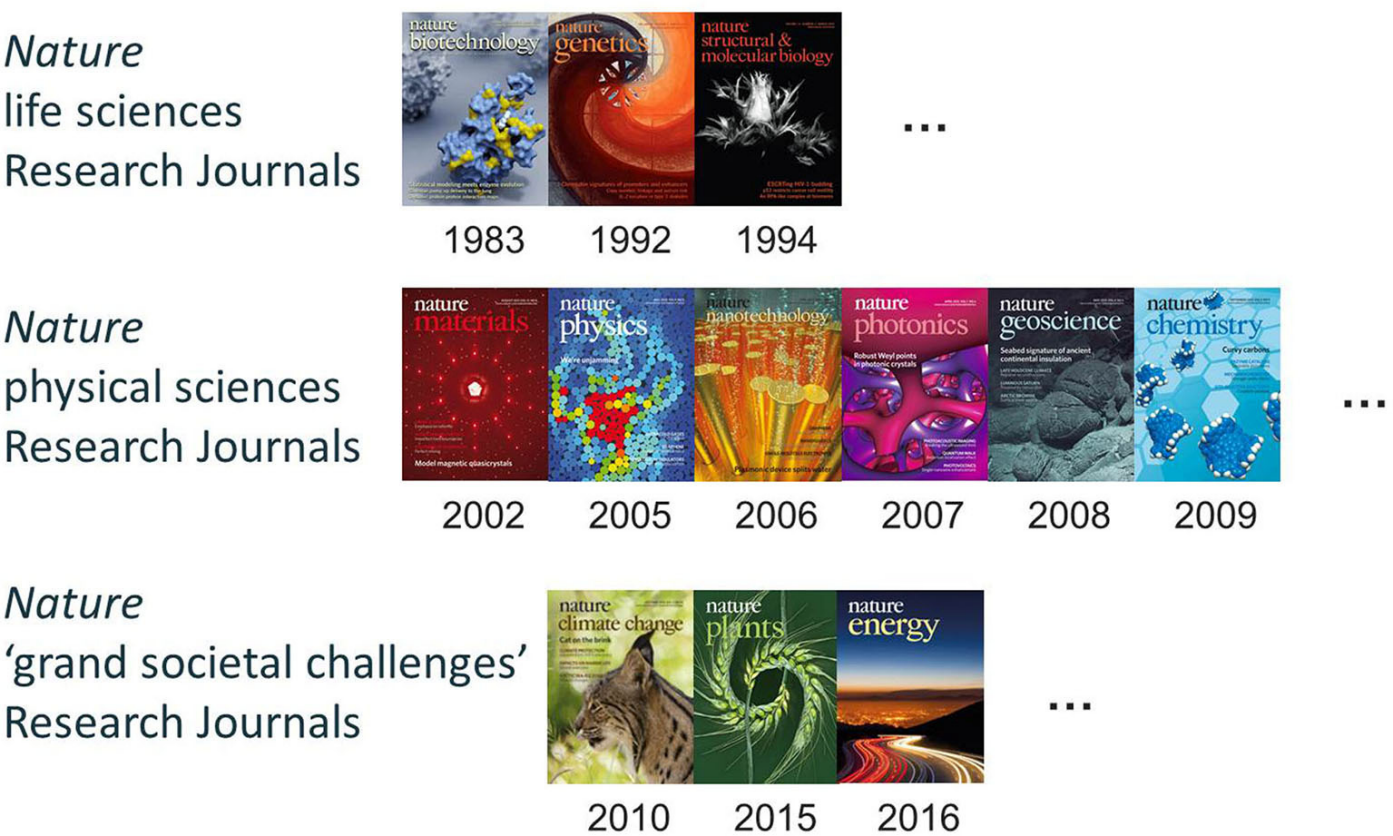
Development of the Nature-branded journals portfolio, adapted from Verberck 2017.^2^

Nature Research's flagship publication is *Nature*, a weekly multidisciplinary journal first published in 1869, whose history has been traced by Baldwin (2015). Nature Research also publishes the Nature-titled research journals (also named Nature-branded research journals, see the chronology on Figure 1), *Nature Reviews* journals (since 2000), society-owned academic journals, and a range of open access journals, including *Scientific Reports* and *Nature Communications*.

As illustrated with the creation of the titles above, in 1983, Nature Research pioneered a commercial strategy to capitalize on the brand names of it most prestigious scientific journals. This has since been repeated by the competing generalist journal *Science*, as well as by journals in medicine, biology and chemistry.

For the past 10 years, *Nature Communications* has been a pioneer within the “Nature family” in many ways. Launched in 2010 as the 17th Nature-branded research title, it was the first to publish content online only (other journals were at this time both in printed and electronic format, some of them still are). It was also the first “hybrid” Nature-branded journal, offering an Open Access option to authors, before flipping to fully Open Access in 2016. One founding principle was to offer a platform for multidisciplinary works, given that the number of multidisciplinary primary research journals was very small at that time. As shown in Table 1, the figures achieved are impressive, which enabled the journal to scale up and expand considerably. Using excerpts from interviews, I will illustrate the place that *Nature Communications* has taken in the general strategy and the development of the Nature Research journals portfolio.

**Table 1:** Nature Communications at 10 (2010–2020), adapted from Infographic: Nature Communications through the years.^3^

**2010**	***Nature Communications* launched as the first hybrid, on-line only Nature journal**
2011	A team of 4 editors, based in London, published 50 articles in the first year
2013	Move to a daily schedule
2014	Flip to fully Open Access at a time where OA content was about 30%
2015	The editorial team grew to 43 strong. The journal continued to publish subscription content that had been submitted prior to 14th October 2014
2016	Content published from January 1st is fully Open Access
2017	Launched “Under Consideration” in support of preprint deposition
2018	100,000th submission (cumulative number of submissions)
2019	34,000 submissions received in the year, over 30,000 reviewers engaged
2020	Editorial team is over 100 strong. 29,621 papers published in one decade

The ACS is a learned society founded in 1876 in New York by a group of 35 chemists. It is a 501 (c) non-profit organization that, as such, is exempt from federal income tax. It was incorporated 60 years later in Washington DC. The promotion of scientific interests through publications is one of the missions described in the charter of the society, which codifies the main operating principles of the ACS.

Today the ACS has about 160,000 members in all areas of chemistry and chemical engineering. It is not only the largest scholarly society in chemistry but also the richest in the world. The ACS is the sole publisher of the Chemical Abstracts database, which is its largest source of income under the name of SciFinder, and one of the top ten science publishers worldwide.

The ACS relies on a collegial governance with multiple bodies (Board of Directors, Council, Divisions, Committees, etc.), and has a considerable number of bylaws and regulations that organize the life of the society. In other words, the ACS is a huge, well-oiled machine. As early as 1908, it organized itself into technical divisions, to enable interaction between scientists who worked or had a common professional interest in a particular topic. The society is currently organized into 32 technical divisions that cover almost all the major sub-disciplines or fields of chemistry, and 186 autonomous local sections. The life of the society is punctuated by two annual meetings (the National Meetings) organized in spring and autumn of each year, and that afford an opportunity for all governance or journal editorial bodies to meet regularly.

(Noel, 2019) offers a chronological description of the ACS Publication Programme ending in 1968, and indicating for each journal a start date, if it has been acquired or separated from the programme, and a title name that may have changed. The ACS created a technical division dedicated to publications in 1969 and now uses the term ACS Journals rather than the Publication Programme. But “rebranding” is not an entirely new strategy, as illustrated by the changes of titles names that have been common throughout the Programme's history.

In 2006, the ACS launched its first ACS-branded journal, ACS Chemical Biology. Since then, around 30 other ACS-branded journals have followed, at a pace of approximately three new journals per year since 2014. From a chemistry perspective, ACS-branded journals explore various scientific and technological “fields” (to quote Bensaude-Vincent, 2018) such as energy, photonics, materials, earth and space, biology and medicine, thus extending the range of topics covered by the initial portfolio. On its website homepage, the ACS emphasizes the vast number of articles (1.3 million) produced and made available in its journals. Its web presence is over 100 pages.

It is therefore this strategy that brings together the ACS and Nature Research, as well as many other publishers (Khelfaoui and Gingras, 2020).

### Data Collection and Analysis

This research is based on the analysis conducted in the course of my dissertation project (nearing completion), the outcome of which can be considered as a composite thesis (Kaltenbrunner, 2015: 23–33). The analyses presented on the following pages emerged from a study of publication practices and strategies of academic chemists confronted with the deployment of open access policies. I conducted more than 80 semi-structured interviews in chemistry departments, libraries and publishers in several countries (France, Sweden, Switzerland, UK, and USA).

Out of this body of material, two interviews with editors at Nature Research were selected as the core material for this study, because they provided detailed insights into editorial activities. I have also included excerpts from written exchanges with the associate editor of a journal led by the American Chemical Society. I have asked the author's permission to publish certain excerpts that have been paraphrased.

Face-to-face interviews with editors at Nature Research were obtained in London at two different times (2013 and 2018). Each interview consisted in three main parts: a biographical narrative, questions about the nature and evolution of their work since entering the publishing industry, and a focus on their practices and how they manage a constantly changing environment. I had a hard time securing the interviews and it was not possible to spend time with the editorial teams. In exchange for this access, the anonymity of the interviewees was guaranteed, which prevents me from giving further details about the editors and the names of their journals. For example, I may not divulge the exact age of the respondents, but I have been able to show a general demographic distribution. I also provide generic job titles and the name of journals (*Nature* X, *Communications* Y, *ACS* Z) to contextualize quotes, while protecting the individual's anonymity. As stated in a tweet issued by the ACS Publisher Center in August 2020^4^, the selection of journal editors is usually a highly confidential process. The profile of the editors and their work environment are listed in Table 2. Emphasis is placed on source protection at the expense of accuracy.

**Table 2:** Profile of the respondents and their work environment.

**Name**	**Martin**	**Evan**	**Andreas**
Collected material	Interview (2013)	Interview (2018)	Written exchanges (unspecified date)
Journal	*Nature* X	*Communications* Y	*ACS* Z
Format	Print/online	Online	Print/online
Journal starting date	2000s	End of 2010s	Unspecified
Position	Chief Editor	Chief Editor	Associate Editor

Interviews lasted for between 60 and 90 min. When the interviewee agreed to be recorded, the interview was fully transcribed and anonymized.

Interview data were coded according to the principles of grounded theory (Charmaz, 2006). I worked with an open coding process (Saldaña, 2013) with multiple successive rounds of coding. This coding was further refined to formulate an analysis of the substance of the conversations with editors and define categories that 1/focus on diverse aspects of their editorial work (“handling manuscripts,” “maintaining the quality of the articles,” “shaping the field”), and 2/reflect the ways in which their journals (as organizations) operate within large publishing houses: “a catalog logic,” “observing each other,” “toward an individualized service.” These themes derived from the data analysis are used in the two sections that present the empirical results below. Some transversal themes (“the curator as an emerging figure,” “editorial innovations”) could have found their place in both sections.

I also interviewed two publishing recruitment specialists at the London Book Fair (2014), to give me an idea of the job market, working conditions, salaries, and so on in the STM publishing industry. They were the managing director and publisher recruitment consultant, respectively, at a small company (11 people) specializing in publishing jobs at all levels. Interviews lasted for 60 min and were fully transcribed and anonymized.

Besides interviews, a document analysis (of editorials, websites, webinars, etc.) has been performed. Since the number of interviews was small, I was careful to supplement the analysis with information collected in Nature Research journals (12 editorials of *Nature X* written in 2013, one editorial of *Communications Y* written in 2018) and on their websites (2013–2019), as well as the min of a webinar (held in 2020) celebrating the departure of the editor-in-chief (EiC) of the *Journal of the American Chemical Society (JACS)* and introducing the new *JACS* editorial team.^5^

Other sources include job offers (7 from ACS, 5 from Nature Research) and CVs, as well as tweets collected at different periods. In processing this collection of texts, videos, images, etc. made available on digital mediums (including the web) and attempting to make sense of the meanings portrayed by these texts or graphical representations, I borrow from the methods of digital ethnography (Hine, 2005; Pink et al., 2016).

Finally, I observed an ACS on Campus event (July 9–10, 2014) jointly organized by the Information Center^6^ at ETH Zürich with the ACS. Organized over two half-days, the event included presentations about scholarly publishing by two associate editors of ACS journals (*Organic Letters* and *Analytical Chemistry*), by a managing editor of ACS journals (on copyright and ethics in scholarly publishing), a career pathway panel including patent examiners and representatives of Swiss chemical industries, and SciFinder training sessions with a regional marketing manager at ACS International Ltd. representing Chemical Abstracts Services, CAS (a division of the ACS).

## Editor's Key Tasks, Respective Role and Relationships With Others

As the consultant working for a recruitment agency explained, half of the people within scientific, technical and medical (STM) publishing have science degrees. This makes STM a particular case in the publishing industry, since elsewhere experience counts more than academic qualifications. Careers are attractive and every advertised editorial position attracts hundreds of applications (more than 10 resumes received on the day of the interview). Entry-level salaries are considered fair (around £20,000 in London) and can rise much higher quickly. After finding a job, candidates are back on the market 2 or 3 years later, as is common in the cultural industries. Within companies, the turnover is high internally [“I'm the only one left from the original team,” (Martin)] because of many opportunities within Nature Research (at the time of the interview, two team members had just left *Nature X* to join other journals). One interviewee moved up the hierarchy of editorial positions before being offered the position of editor-in-chief.

The editors on whom this work focuses are in their forties (between 40 and 49 years old). All have a PhD and postdoctoral experience in European universities (France, Germany, UK). For both Martin and Evan, a career in publishing was more of a default choice: they had limited motivation for editorship, which is consistent with the perception of this occupation (ensuring the “mechanics” of the editorial work) in academia.

“*I've always kind of known publishing is a career but I wouldn't say when I was training as a scientist, my dream was not necessarily to work in publishing, I think like probably most people in this building actually.” (Evan)*

### Expected Skills for Entry Into the Occupation

At Nature Research, the hiring procedure includes an interview, a reading and writing test, and a kind of role play. Ten years apart, the hiring process was the same for Evan and for Martin: the recruiter tested the candidate's ability to write quickly and under pressure.

“*We look for people who can get to the bottom of a paper quite quickly even if they don't necessarily understand it and, to some extent, we look for people that can cope under pressure because this is a relatively high pressure job, you can't control the workflow so, you know, if 30 people decide to submit 30 papers in your area in one week, I mean, that's an extreme example, but you know just before Christmas, just before Chinese New Year, lots of work comes in because every, all the authors submit before they go on holiday.” (Evan)*

As the manuscripts submitted during the test do not present any particular problematic issues, candidates are expected to be flexible (neither arrogant nor naïve) as there is no single answer at this stage of the process, to be able to put themselves in the position of an editor and not an author as they were before, to take a critical look at the reviewers' reports, and so forth. These skills are tested to see if the person is capable of developing arguments. All these skills are action-oriented; they test sound decision making.

“*You know, because you don't really know how to be an editor until you start being one, but some people have perhaps slightly more inherent skills that make them more suitable for the job or you know, they just develop them by an inventive approach.” (Evan)*

“*Again, it's looking at whether or not they can assess reviewers' reports like an editor, rather than like an author. You know, can they understand what's important about the reviewers' reports and see what the reviewers are either clearly saying or saying between the lines? And make sensible decisions.” (Evan)*

In terms of identity, these excerpts attest to the necessary transition from “being an author” to “being an editor” and the inventive approaches that may underlie this latter posture. As a member of the professional group of editors, Evan describes the foundations of his legitimacy, and thus his autonomy, by drawing on values such as “a high pressure job,” which doesn't stop between Christmas and New Year's Eve and validates the work of the reviewers in a dialogue with them.

Editors' social skills do not consist only of their ability to assess but also to attract potential manuscripts. They are also closely linked to community building, as we will see next.

### Modalities of Work and Organization of the Editorial Process

At *Nature X*, Martin leads a small team (5 people at the time of the interview, 3 different European nationalities) of senior or associate editors covering topics in their field of expertise (physics, physical chemistry, biology). The team is cohesive (“we know each other well”) and they meet every 2 days.

In terms of supervision, “the journal is in a way a direct extension of the laboratory, where newcomers gradually gain in autonomy through contact with the most experienced” (Evan). Martin's team handles around 250 manuscripts per month, or an average of 12 manuscripts per week and per editor, for which a report is written. Forty percentage of the manuscripts are rejected outright, 20% are sent to reviewers. The journal receives “many, even too many papers. They come to us.” At the time of the interview, Martin had been asking for another person for 3 years, “maybe I'll get him/her this year.”

At *Nature X*, the range of fields covered is wide: “some of the articles published are in very fundamental physics or fundamental biology.” Martin is committed to respecting diversity and finding a balance between all areas across the journal's cover spectrum. He quotes the example of an article published in a subfield that he finds “old-fashioned but where interesting things happen.”

This is also the case at *Communications Y*. As the scope of the discipline is broad, the journal is open to all research that may be at the cutting edge of chemistry.

“*We've been relatively flexible, as a journal, about what we consider to be sort of the edge of chemistry. So our philosophy really is: if authors have decided to submit to us and we can imagine that maybe some chemists can be interested in this paper for a reason that has to do with chemistry, that's kind of enough in terms of scope.” (Evan)*

One informant detailed the organization at *Nature Communications* (2012–2017). He described a considerable change in 5 years, where the journal had become a very large structure with more than a 100 editors. Again, it is helpful here to think in terms of the journal as an extension of the laboratory and as “a collective workshop,” to use Shapin's (1994: 367) words, a site at which labor is increasingly finely divided.

“*When I joined, it was a small structure. There was a chief editor and then some editors. And now they have like a hundred full-time editors. So within that there is a management structure. So I was a team manager. So there was the chief editor, the chief of physical sciences and then me and I was running a team of editors, they were covering basically my area plus inorganic, physical, materials, nano, energy, catalysis. And I had editors in Shanghai, New York and London, and that was just because that was where the editors with that expertise were based.” (anonymous informant)*

### Handling Manuscripts

Evan estimates that tasks of all kinds around the manuscript (“all the sort of manuscript's life”) account for about 80% of his total working time:

“*In terms of how you spend the day, it's largely handling manuscripts. That's the sort of major task of an editor. So all the kind of processes of the lifecycle of a manuscript, it varies slightly depending on how selective the journal is.” (Evan)*

In terms of work sequences, the first is triage: deciding whether or not the manuscript is sent to reviewers. This involves knowing the literature covered in the paper, which means keeping up with articles in the field. Martin also insists on the importance of the state of the art (“we keep up with the current literature and developments, we have time to read”).

**Figure 2:**
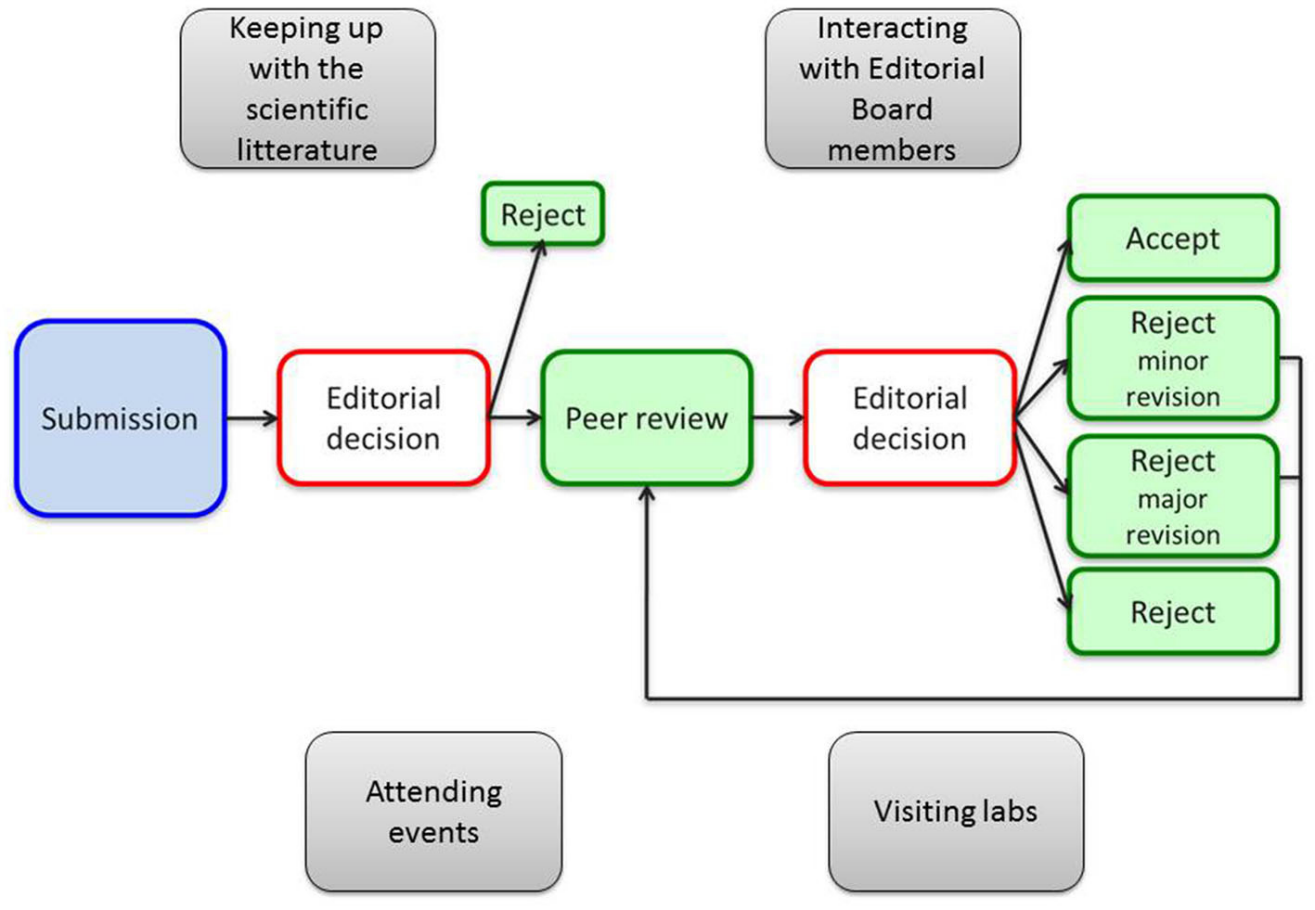
Editorial process at Nature Research journals. Initially adapted from Verberck 2017^2^, enriched by the author with a set of complementing tasks (in gray).

While the synoptic diagram shows the key phases of editorial decision making (in red in Figure 2), it must be enriched by a set of tasks that complete the editing activity: keeping up with the scientific literature, attending events, visiting labs, interacting with editorial board members, and so on.

In his role as editor-in-chief, Evan does little copy editing or proofreading. The strategic choice of outsourcing proofing in low-income countries is shared by many publishers (Horbach and Halffman, 2020) but also reflects the hierarchy within the Nature Research journals portfolio and the human resources that are allocated to them:

“*The more selective journals here, so* Nature *and* Nature Research *journals, they have full-time copy-editors. Their jobs is to copy edit entirely, that's why if you read any* Nature *paper there's always been a slight like “house style*,” *regardless of who the authors are, because they work closely with the copy-editors. At* Nature Communications *and* Communications Y, *we're not quite resourced within that way, so it's the editors that do it and it's a lightly touch, really. So we will always make sure that the title and the abstract are very very accessible, we'll do kind of a light copy-edit of the introduction and the conclusions, the things like the results and the methods, we'll give the authors recommendations and we point them on online tutorials which are free, editing services that they can pay for, we have owned a company but obviously other ones are available.” (Evan)*

At the ACS, key phases are all deeply embedded in the society; even if their editorial policies differ, the ACS journals share the “issue publication information,”^7^ and the same editorial process within the framework of an organization (the Publications Division) that publishes a weekly magazine (Chemistry & Engineering News, C&EN) and the 50 scholarly journals focused on chemistry and chemistry-related topics. Editorial production activities are handled in Columbus, OH.

**Figure 3:**
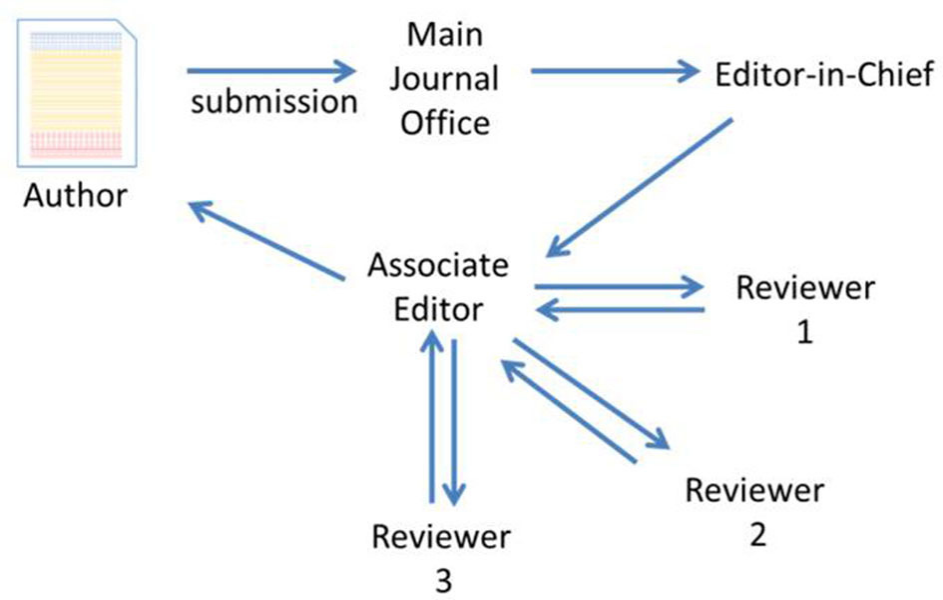
Editorial process at ACS journals (adapted from the presentation of E. Carreira, ACS on Campus at ETH Zürich, July 9, 2014).

In terms of peer reviewing, what Figure 3 suggests is concretely described in the exchanges with Andreas: even if the level of collegiality is high, there is the hierarchy between the EiC and the associate editor(s). Andreas was associate editor for 10 years in one journal before he was offered the position of EiC in another. Assuming there are approximately a dozen tiers of associate editors at *ACS Z*, he stresses the need to enforce the communication between associate editors (more frequent, substantive, cohesive, etc.) and proposes that the meetings take place during the ACS National Meetings.

As Evan sees it, the proposed paper has to satisfy the criterion “enough or sufficient advances beyond the state of the art,” which is at the discretion of the editor, as illustrated here:

“*They are two fundamental tasks I would say. So, manuscripts come in, and you decide whether or not they're going to go out for review for that journal. […] So looking at the other kind of relevant recent articles in the field, deciding where the state of the art is, and then deciding whether the manuscript makes enough or sufficient advances beyond the state of the art that is of interest to the readership of that journal.” (Evan)*

This criterion “enough or sufficient advances beyond the state of the art” also depends on the journal. For *Nature Communications*, “the advance doesn't necessarily need to be enormous, it's quite a selective journal, but yeah it's not super difficult to get into,” whereas for *Nature Y* “[the authors] might need to say ‘new chemistry.” (Evan)

Both Martin and Evan are keen to affirm the separation of scientific and commercial functions within the Nature Research group. “Priority goes to the articles we publish.” (Martin). “All the editors here just assess the science.” (Evan)

“*Then you manage the manuscript all through the review process, so this thing like chasing reviewers is all automated. The reviews go out, the reviews come back in, you make a decision for the manuscript, being rejected or accepted.” (Evan)*

Traditional ways of organizing the editorial process still prevail, despite evidence of flaws in old practices and the proposed advantages of new ones (Horbach and Halffman, 2020).

“*So, all of this goes through a kind of on-line system, however the tasks are really the same as they were 100 years ago when people were posting papers to each other.” (Evan)*

### Maintaining the Quality of the Articles

As head of the journal, the goal of the EiC is to create the conditions that will enable publication of high-quality papers: “Our objective is to maintain quality” (Martin). This is crucial for the survival in the competitive space of Nature Research's journals.

“*My personal feeling is a journal operating in this space will kind of live or die on the quality of its research content.” (Evan)*

Key criteria concern novelty (“we judge novelty“) and completeness of the work, even if it means (sometimes) “losing” the paper. Martin distinguishes two categories of papers: “the papers that are good and the ones that are exceptional.”

“*I want the most comprehensive works.” […] “It's nice when the papers come back to us after a year and a half with the answers to the questions.” […] “Sometimes the papers go somewhere else, as a result.” (Martin)*

It is also a matter of guaranteeing the frequency of publication and ensuring that a minimum number of manuscripts are being processed. For instance, Evan does not want to commit to writing editorials in the launch phase of the journal; he is almost sure he would not be able to keep up, which would throw his entire team's workload off balance.

In terms of conceiving of what a journal is, we are a long way from the journal club (Topf et al., 2017) where the article is discussed with peers. When I ask Evan about an article already published in *Communications Y* that he is proud of, he takes the example of a paper describing a molecule with special properties that was voted “molecule of the week” at the Cambridge Crystallographic Data Center (CCDC) and is featured in C&EN, the official organ of the ACS. From Evan's point of view, the article is a finished product, the ideal trajectory of which is to end up in a textbook (Brorson and Andersen, 2001). It adds to a broad and consensual knowledge base and contributes to the cumulative growth of knowledge in chemistry.

When I ask the same question to the editor of another ACS journal (recently launched) on Twitter^8^, he tells me that there are several answers: the first (photocatalysed hydrolysis of arylethers) is “very cool,” the second (catalytic hydrogenation of polyurethanes) “promises a lot in terms of applications,” the third is a Perspective paper on the cornucopia of cyclic allene chemistry. The examples are all associated with graphical abstracts (a visual summary of a scientific paper that appears on a journal's table of contents).

As soon as papers are published in *Communications Y*, the team tries to amplify the content on Twitter, especially if one of the authors is active on that medium. Within the Nature Research chemistry community, there is an outward-facing website group (“it's kind of like a Facebook page, all the chemistry editors are on it,” Evan), which many authors join to write what is called a “Behind the paper.^9^”

“*So it's kind of a... I don't even think it's got a DOI like a blog post but it's given sort of the story of how [the authors] did the research or why they've approached a particular research question or topic, you know, we retweet and link to things like that as well. So, just trying to add value to the papers where we can.” (Evan)*

Surprisingly, one can learn in a “Behind the paper” that was published recently that an author had a colleague who had a press release of his research results published in *Communications X*, and he also wanted a press release for his own results in *Communications Y*. We are far from the gatekeeper exerting considerable control on the scientific discourse; this exchange resembles the kind of reciprocal gift-giving described by Mauss (2006), as part of a service toward the author.

### Shaping the Representation of the Field, in Powerful Associations With Scientists

A key aspect of the job is also liaising with the scientific community through laboratory visits (about once a month, Martin), where researchers can pitch their work or ask questions. Editors also attend international conferences to advertise journals and encourage submissions:

“*So we attend conferences, very big conferences like the ACS and Springer Nature will have a booth, you know, in this sort of expo we'll spend some time at the booth, people come and see us. We'll try and attend some talks just to get a flavor of you know, what is the most exciting field.” (Evan)*

In both set-ups, publishers partner with institutions to offer events that a university or department or lab group can host free of charge or can purchase, with workshops covering all aspects of manuscript preparation. Nature Masterclasses consist of a 1- or 2-day workshop, attended by two journal editors from Nature Research, with an audience of about 25-30 researchers in each workshop. The (paid) service includes one-to-one interaction with editors and an opportunity for abstract review.

ACS on Campus programmes range from 90-min webinars to full-day events^10^. The program for the free event observed at ETH Zürich in 2014 was specifically designed to bring together journal editors who are professors at ETH, ACS representatives, career consultants and local chemistry professionals, patent examiners and librarians. Students and faculty members were invited to attend the seminar, which was publicized by posters and flyers placed in departments buildings (free food and give aways were also provided). Entitled “Let us help move you forward. Get published. Find a job. Get the skills you need,” it covered a wide range of topics (publishing, career paths and chemical information management) and was attended by 80 persons, one third of whom were PhD students. In terms of scientific publishing, journal editors took the opportunity to orally deliver key messages or advices (for instance “I don't want my reviews to be publicly available,” “I don't pay attention to nationality,” “a full time PhD student looks at the Supplementary Materials” etc.). Andreas also attests to the interest of these joint events in gathering the needs of researchers (the SciFinder training sessions at ETH were designed based on responses to a SurveyMonkey questionnaire) and promoting mutual understanding:

“*I have represented [ACS journals] several times at ACS Campus events and always got good feedback. The more authors/readers know about us, the more transparent our handling of articles becomes.” (Andreas)*

At *Communications Y*, there is also an endeavor to work collaboratively with Editorial Board members on some of the papers. I will detail this point in the next section.

## Hierarchies and Dependencies Within Publishing Houses

### A Catalog Logic

In both publishing houses, the periodicals are locked into a catalog logic where the journals are not necessarily competitors and have an assumed place and hierarchy. *Communications Y* is described as “a particular publishing space” which does not compete with *Nature Communications*.

“*While there is some conversation between* Nature Communications *and my journal*, Nature Communications *is a more selective journal, you know, we exist because they are more selective. So my aim isn't really to like get a higher impact factor than* Nature Communications. *It's to sort of give the authors that come to me the best possible service, to try and publish the best papers that I can in this particular publishing space.” (Evan)*

Relations seem to be fairly peaceful within the vertical chemical “silo” (with its asserted hierarchy) at Nature Research, but a form of “friendly” competition nevertheless exists between editors of chemistry journals at Nature Research. Evan describes the case of manuscripts sent to *Nature Communications* whose authors did not aim high enough and which he kept for “his” journal.

“*I've had some papers come to me when I was at* Nature Communications *for example, and I thought: oh, if the authors had tried, this could maybe have got into* Nature Y*! But your job is to publish the best you can for the journal and to give the authors the best service you can, so I've always kept the papers myself.” (Evan)*

The hierarchy within the group is also reflected in the availability of human resources to write important pieces: editorials.

“Nature *publishes at least 2-3 editorials every week because it has staff who do this*, Nature Y *they publish one a month because they have kind of staff resources*, Nature Communications *for a long time didn't publish because they were too busy. Now, they finally kind of, they've got the appropriate number of editors and a little bit more time, and the journals are more stable. So they are writing more editorials.” (Evan)*

“*We have lots of kinds of guided transfers […] so all those journals have chemistry editors. So the chemists get together once a month and we discuss things like: chemistry policy, reproducibility issues, but also the respective bars of each of the journals. So hopefully if Nature Y are rejecting a paper they'll have a good idea about whether it will be a good potential fit for us or for Nature Communications and I can recommend the authors […]. And this is a thing that like the ACS, the RSC, most publishers do this.” (Evan)*

As these two interview excerpts show, being an editor also means writing regular editorials in the journalistic sense, i.e., producing a discourse comprising norms, values and worldviews that define what is appropriate for an individual to be considered a competent and recognized member of this professional community.

### Observing Each Other, Trying to Distinguish Each Other Through Formats

All EiCs have competing or partner publishing houses as reference points. For instance, Evan knows how editors are recruited at the Royal Society of Chemistry (RSC), whose offices are based in Cambridge (UK), because he took the tests himself. He also knows the salary range of his competitors.

“*When I interviewed at the RSC, for example, the test was very different, I was given a reading comprehension task. I was asked to do some copy … it's quite a different way of approaching [here].” (Evan)*

Martin finds that the competition is more internal to Nature Research than with the outside world. It is rather toward its sister and brother journals that he looks. But he admits that working in a big publishing set-up like ACS or the American Physical Society (APS) does not appeal to him (“it's too large”).

Because there is a clear overlap concerning topics covered by different ACS journals (one of which is quite close to *ACS Z*), Andreas expresses the need for clear guidelines. According to him, formats are a simple way to differentiate journals. By specifying what is expected in terms of format (for instance a letter with a few printed pages, a full paper, etc.), the journal can better orient the authors on what is expected of them.

On the occasion of the departure of Peter J. Stang who was the editor-in-chief of the *Journal of the American Chemical Society* (its “flagship” journal) for almost 20 years (2002–2020), his successor recalls the editorial innovations of all kinds implemented during his tenure:

- Graphical abstracts (2002)
- Virtual issues (2008)
- Facebook (2008)
- Twitter (2009)
- Cover art (2009)
- Perspectives (2009)
- Mobiles (2010)
- Spotlights (2012)

Finally, insofar as *Communications Y* is relatively recent, the journal is trying to get into a space where there are many other “good chemistry journals.” Paying for papers with reasonable APC serves as a proxy for ascertaining what is truth and valuable.

“*At Communications Y, we're trying to operate in a space which isn't really particularly well served by Springer Nature at the moment but it's where lots of other good chemistry journals operate, you know, like [name of a journal] that is [amount of the Open Access fee in pounds]. I think what we're publishing is more selective than [name of a journal]. So, you know, the more selective a journal is, kind of the higher the APC has to be, because you're paying for all the rejected papers. I think the author service that my team and I are adding is comparable actually to what [name of a journal] are adding and with half the price.” (Evan)*

### Times Are A-Changing: The Emerging Figure of the Curator

As editor, Andreas projects himself in the role of a curator of reviewing more than “the sole gatekeeper to publication,” claiming that the worst scenario that can happen to journals is when authors and readers consider them to be controlled by a “mafia-type” organization.

According to him, “*ACS Z* should focus on being a ‘society journal' from scientists for other scientists, different than commercial publishers.” Again, the reference is the other, without knowing very well what the term “commercial” covers. Actually, all Associate Editors have a work contract with ACS, which is also the case at the RSC. Since some editors review a lot of manuscripts and others not so much, Andreas suggests that the number of handled manuscripts should be taken into account in regard of the paid compensation.

The approach taken at *Communications Y*, as with all the *Communications* journal series, is that of a shared editorial model with in-house editors working alongside an Editorial Board model consisting of active researchers. The collaborative Editorial Board is conceived of here as an intermediation authority between science and decision making and, above all, a way not to be disconnected from academic research. At the time of the interview, “Editorial Board members haven't really pushed many papers through the season:”

“*We were hoping the people that we worked with would actually have time to do some editorial work and to benefit from it. So they'd learn how selective journals handle papers. You know, hopefully that would feed back into their own submission to other selective journals and it's a chance to sort of, you know, almost like have a peek behind the curtain of how stuff works here, to work with people that have, you know, working in kind of a Nature environment. We thought maybe it would be attractive to some researchers.” (Evan)*

A strong wish of all EiCs is to include young scholars in the editorial process. For Andreas, it is really essential to attract junior editors into the Editorial Board. That means training them, letting them see what is “behind the curtain,” without which the loss of confidence among young researchers will be total.

The editor succession is also a political and representational issue. In June 2020, Thomas Hudlicky, professor of chemistry at Brock University, Canada, published an essay in *Angewandte Chemie International Edition* discussing factors influencing the progress of organic synthesis over the past 25 years in a way that many found offensive, considering offensive opinions about women and other underrepresented groups in science were being expressed, and that the Chinese research community was being unfairly denigrated. Following a deluge of criticism on social media, the article was withdrawn and completely deleted from the Angewandte publication records by the editor-in-chief. The editors themselves acknowledged a “breakdown in editorial decision-making” and two of them were suspended. In protest against the publication, several members of the International Advisory Board resigned while some authors publicly announced that they would no longer publish in the journal. In an editorial, a group of six chemists expressed their deep concern “that the appearance of this Essay has harmed the trust of the scientific community in the journal's procedures to select essays of the highest scientific standard without bias” (Beck-Sickinger et al., 2020). The journal began an internal investigation, the effects of which are still difficult to measure (only 1 year and half has passed).

### Toward an Individualized Service for Each Author and Subscriber

In 2013, the horizon already indicated an individualized service to each author and subscriber. Martin points out the huge investments in software, for instance Springer Nature's acquisition of the Figshare platform. There are more and more computer scientists in the company, which “marks the shift to big data.” He appeals to journalists and obtains their help for the press releases, but he does not intend to publish more, which validates the logic of scarcity emphasized by the detractors.

## Discussion

As shown from empirical evidence above, the general mechanisms at play between chemistry journals oscillate between competition and cooperation: in both cases, these mechanisms intersect or even combine. As Musselin pointed out (2018), the issue of cooperation among competitors is well developed in the seminal work of Harrison White based on in-depth case studies of American firms (White, 1981, 2002). In one of the three strategies identified by White, competitors are engaged in a competition for quality. In order to compete, firms need to identify their competitors, observe and learn about them, emulate them, etc. This can lead to the production of a certain social unity because competitors share common norms and the social regulations that follow from them, such as—in the case discussed in the article—belonging to a scientific/learned society, participating in conferences, etc. Alliances of competitors belonging to the same category may thus emerge in the form of what White has called “market segments” (White, 1981). In his empirical study of restaurants in the North of France, Eloire (2010) observed that only “gastronomical” restaurants developed cooperative behavior, compared to other restaurants that have price-driven strategies. Building on White's work, he argued that it was precisely the type of competition in which gastronomical restaurants was engaged (a competition for quality, in which quality matters much than price) that explained the development of cooperation. The importance of cooperation among competing chemistry journals might therefore be explained by the fact that, as for gastronomical restaurants, they compete for quality on a market segment that is disciplinary.

Like all forms of competition, competition among chemistry journals relies on classifications of all sorts (Fourcade and Healy, 2013). How do editors handle metrics and indicators? As noted above, the hardest part for *Communications Y* is to establish oneself in the publishing landscape. Evan had been thinking in terms of numbers of papers published during the launch phase that lasted almost 3 years. At the time of the interview, he felt more serene at the end of this “probationary” period because the journal was almost guaranteed to grow, due to the broad spectrum it covered. He explained that there is a lot of internal discussions (including with the upper management) that goes on about launching, and Nature Research doesn't really launch journals that fail (failures are exceptional or non-existent).

In 2013, Martin admitted to being poorly equipped with metrics (a subject on which he communicates little or never in editorials). With one exception, Impact Factors were not mentioned in the interviews. This is consistent with a general observation about chemists' (non)use of impact factors in interviews. The market agencement of “elite” chemistry journals is relatively circumscribed; as exemplified in the empirical material, actors know their competitors perfectly.^11^

The entry through the challenger organizations is a dynamic framework which, coupled with the study of professional trajectories, allows us to define the social skills (in the sense defined by Fligstein (2001) as “the ability to engage others in collective action”) developed by editors at Nature Research. Evan's career path is emblematic as he moved through different journals at Nature Research and then launched a new journal that he describes as a “particular publishing space.” In the important and regular documents that editorials are, editors produce meaning for others, because by doing so, they produce meaning for themselves.

My purpose in this work is to use a set of conceptual understandings, such as social skills or market agencement to avoid addressing journal editors, researchers and librarians as three separate silos.

The empirical description detailed above shows that journal editors shape the representation of the field in powerful association with scientists who, in turn, publish in ACS, Springer Nature, RSC, or Wiley journals, depending on the nature and often contingency of the collaboration with others. This representation of the field, and the norms attached, is also constructed in a rather strong relationship with librarians, as illustrated by the set-up of the ACS on Campus event. In market agencements, innovation is the driving force of market evolution in which skilled social actors play a crucial role.

The practices that are described here seem to be quite general. What is specific to chemistry? Part of the cultural authority of science derives from its ability to guard its boundary (Gieryn, 1999). In a study contrasting biology and chemistry regarding laboratory safety regulations, Silbey documented the way chemists have taken on the role of regulating themselves and mobilize their disciplinary authority to the benefit of collective identity-based actions (Silbey, Forthcoming). When it comes to chemical substances and products, but also to publishing, chemists rely on historical relationships with government regulations (the history of *JACS* is marked by massive imbalances caused by changes in scientific, institutional, and regulatory environments, cf. Noel, 2020). In 2017, three learned societies (the ACS, the Royal Society of Chemistry and the German Gesellschaft Deutscher Chemiker) succeeded to launch, then manage, the preprint server (ChemRxiv) “designed specifically for the global chemical sciences community.” They became partners in regulation.

## Conclusion

In this work, I have explored the large range of activities covered by editors-in-chief in two publishing houses. As shown by the pioneering work of Horbach and Halffman (2020), journals are not edited just by their editors-in-chief and do not amount to just the editor(s)/reviewer(s) duo, given that the editorial process is hierarchically structured to a large degree, with distinct tasks for distinct layers of the process and thereby a clear division of labor among these layers.

The empirical evidence reveals a wide palette of practices that refute the metaphor of “editors as mere gatekeepers.” Starting from a description of their tasks, role and identities, the theoretical framework adopted (the description of a market agencement) allows for a more complex description of their functions. A term like “gatekeeping” places too much focus on the editorial process often reduced to peer reviewing, which means that we lose an understanding of the social and technical complexities that come with the editorial role. Depending on their degree of involvement in one or the other activity (handling manuscripts, managing a team, assessing articles, covering the field and shaping its representation, and so on), editors may be seen as planners, brokers or facilitators, curators, gatekeepers… or something of everything at the same time.^12^ To varying degrees, journals are described here as an extension of the laboratory.

The article is based on original documentation and attempts to parallel publishing spaces that are generally considered opposable (commercial *versus* non-commercial).While a commercial strategy exists at the ACS (and is effectively translated into a manuscript transfer system), in practice it is deployed with multiple decision-making paths that also involve humans (editors as well as authors, staff of the publishing house and so on), technical devices, classification algorithms and so on. The purpose of the article was to highlight similarities between publishing houses. The interviews underline the feeling or need to belong to a “family,” whether disciplinary or corporate. In what seems to be a race to create and differentiate more and more journals, the actors observe each other and try to distinguish themselves through formats. The strength of the organizations described lies in their long history of experimentation and failure, even if the latter is not emphasized in the editors' narratives.

This paper deals with the case of two publishing houses anchored in countries that historically are home to publishing and chemistry. It is based on a limited number of interviews examined in detail, which are enriched by sources of all kind. Are these results generalizable to other journals in the same set-ups or published by other learned societies or private publishers? While academics often focus on the dark corners of how a journal works, I argue we would learn more by looking how these assemblages of people, knowledge, technology and sites act collectively. In the field of chemistry, expansion to other contexts or types of journals would undoubtedly afford interesting points of comparison.

## Data Availability Statement

The datasets presented in this article are not readily available because the anonymity of the interviewees was guaranteed, which prevents me for giving further details about the editors and the names of their journals. Emphasis was placed on source protection at the expense of accuracy. Requests to access the datasets should be directed to marianne.noel@univ-eiffel.fr.

## Ethics Statement

Ethical review and approval was not required for the study on human participants in accordance with the local legislation and institutional requirements. Written informed consent for participation was not required for this study in accordance with the national legislation and the institutional requirements. Written informed consent was not obtained from the individual(s) for the publication of any potentially identifiable images or data included in this article.

## Author Contributions

MN: conception and design, data collection, analysis of the results, and writing.

## Funding

Part of research reported in the article was funded by the Agence Nationale de la Recherche (ANR-09-SOC-011). This work has also received traveling support from the Institut Francilien Recherche Innovation Société (IFRIS).

## Conflict of Interest

The author declares that the research was conducted in the absence of any commercial or financial relationships that could be construed as a potential conflict of interest.

## Publisher's Note

All claims expressed in this article are solely those of the authors and do not necessarily represent those of their affiliated organizations, or those of the publisher, the editors and the reviewers. Any product that may be evaluated in this article, or claim that may be made by its manufacturer, is not guaranteed or endorsed by the publisher.

## Footnotes

^1^PhD-level scientists with research experience who have already published.

^2^ https://nauka.kz/upload/files/Publishing_in_Nature_Research_Journals.pdf

^3^ https://promo.nature.com/ncomms-10yr-infographic/?utm_source=nature&utm_medium=referral&utm_content=null&utm_campaign=NCOM_1_SZ01_GL_10yrs-sit

^4^The selection of ACS journal Editor is typically a highly confidential process. In a new blog, Chair of the search committee Nicole S. Sampson offers a unique insight to the search of a new Editor-in-Chief for @J_A_C_S and JACS Au.

^5^ https://connect.acspubs.org/jacs-special-event?utm_source=pubs_outreach_marketing&utm_medium=linkedin&utm_campaign=PUBS_1220_JHK_JA_jacsat_JACS&ref=pubs_outreach_marketing

^6^The Chemistry-Biology-Pharmacy Information Center at ETH Zürich is a joint facility of both the Department of Chemistry and Applied Biosciences (D-CHAB) and the Department of Biology (D-BIOL).

^7^ https://pubs.acs.org/doi/pdf/10.1021/jav144i002_1547961

^8^ https://twitter.com/MNoel75/status/1414977104182140928

^9^ https://chemistrycommunity.nature.com/channels/1465-behind-the-paper

^10^ https://acsoncampus.acs.org/

^11^In a published editorial, Martin extracted the number of research papers per months published by *Nature X* and 6 of its competitors to illustrate the growth of open access publishing.

^12^Martin confessed he was a bit atypical as he still reviewed papers, which was less and less the case for other EiCs.
